# Seasonal changes in positive airway pressure adherence

**DOI:** 10.3389/fmed.2024.1302431

**Published:** 2024-02-16

**Authors:** Arnaud Prigent, Clément Blanloeil, Dany Jaffuel, Anne Laure Serandour, Franck Barlet, Frédéric Gagnadoux

**Affiliations:** ^1^Groupe Médical de Pneumologie, Polyclinique Saint-Laurent, Rennes, France; ^2^Centre du Sommeil Polyclinique Saint Laurent, Rennes, France; ^3^Elia Medical Ouest, Cesson-Sévigné, France; ^4^Département de Pneumologie, Hôpital Arnaud de Villeneuve, CHRU de Montpellier, Montpellier, France; ^5^INSERM U1046 – CNRS 9214 – Physiologie et Médecine Expérimentale Cœur et Muscle, Université de Montpellier, Montpellier, France; ^6^SLB Pharma, Rennes, France; ^7^i-GEIA 14 rue Pierre Grenier, Boulogne-Billancourt, France; ^8^Service de Pneumologie, CHU d’Angers, Angers, France; ^9^INSERM, CNRS, MITOVASC, Equipe CarME, SFR ICAT, Université d’Angers, Angers, France

**Keywords:** obstructive sleep apnea, positive airway pressure, adherence, seasonal changes, climate, temperature

## Abstract

Through their effects on sleep duration, bedroom environments, and pollen allergies, seasonal variations may impact positive airway pressure (PAP) adherence. We analyzed daily PAP telemonitoring data from 25,846 adults (median age 64 years, 67.8% male) treated with PAP for at least 4 months [mean (standard deviation, SD) duration of PAP: 5.5 years (SD 4.1)] to examine seasonal changes in PAP adherence, leaks, and residual apnea-hypopnea index. We demonstrate a significant decrease in PAP adherence in June compared to January (mean (SD): 0.37 (1.54) h/night) that achieved the minimal clinically important difference (MCID) of 30 min in 13.9% of adults. Furthermore, we provide novel data supporting the association of rising temperatures with seasonal changes in PAP use. Indeed, the most pronounced decline in PAP adherence was observed during the hottest days, while PAP adherence was only slightly reduced during the coolest days of June. Clinicians should be aware of seasonal changes in PAP adherence that are likely to be exacerbated by climate change.

## Introduction

Compared to no treatment, positive airway pressure (PAP) therapy for obstructive sleep apnea (OSA) is dose-dependently associated with a reduction in excessive daytime sleepiness, impaired quality of life, motor vehicle accidents, and blood pressure (1). Multiple factors affect PAP therapy adherence, including socioeconomic status, psychological factors, OSA severity, side effects, and discomfort (2). Through their effects on sleep duration, bedroom environments, and pollen allergies, seasonal variations may also impact PAP adherence. This study aimed to examine seasonal changes in PAP adherence and the association with temperature variations. We hypothesized that daily PAP use could drop in June compared to January and that this decrease in PAP adherence could be linked to the rise in temperatures.

## Methods

The e-QUALISAS study analyzed 1-month de-identified telemonitoring PAP data from a unique home-care provider (ELIA Medical) database in January, June, and December 2021, including daily PAP use, device-reported residual AHI (AHI_PAP_), and 95th percentile non-intentional leaks. All adults were above the age of 18 years, started PAP before September 2020, and were treated for at least 4 months before the beginning of the study. All data were collected using the PAP software (AirSense 10, ResMed, Australia). For each patient, monthly averages of PAP adherence, AHI_PAP_, and 95th percentile non-intentional leakage values were calculated. Daily PAP use (h/night) was considered the use during the 24 h. Age and sex were also available in the database. Using June 2021 MeteoFrance data, the daily minimal temperature in 23 cities was collected. The healthcare provider has 23 agencies spread across France. For each adult, the minimal daily temperature of the city (or mean of cities) that best matched the area where the adult lived was considered. All included adults gave their informed consent for data collection and anonymization. The study had been registered on the Health Data Hub platform (N° F20220715144543).

For statistical analyses, continuous data were expressed as mean (standard deviation, SD) or median [interquartile range IQR] according to a normal or non-normal distribution. The Friedman test was used to compare paired medians. To measure the strength and direction of the association between variables, Pearson’s and Spearman’s tests were conducted according to the normal or non-normal distribution of data. For each patient, daily PAP use and their area of residence were recorded. Using the location data, daily temperatures were obtained from the nearest weather station. Consequently, a large matrix was compiled comprising, for all patients, the date, daily temperatures, and PAP use. Data were analyzed using a linear mixed-effects model for repeated measures, in which patients were considered the random variable. Python was used for data processing, statistical analysis, and graphical visualization. The packages pandas (v2.0.3), NumPy (v1.24.3), and SciPy (v1.10.1) were used for data processing. The packages stats (v0.1.2a0) and statsmodels (v0.14.0.) were used for statistical analysis and model fitting. The packages matplotlib (v3.7.1), seaborn (v0.12.2), and plotly (v5.14.1) were used for data visualization.

## Results

Data from 25,846 adults (mean age: 63 (12.6) years, 67.8% male) treated with PAP for at least 4 months (mean duration of PAP: 5.5 (4.1) years) were analyzed in the present study.

**Figure 1:**
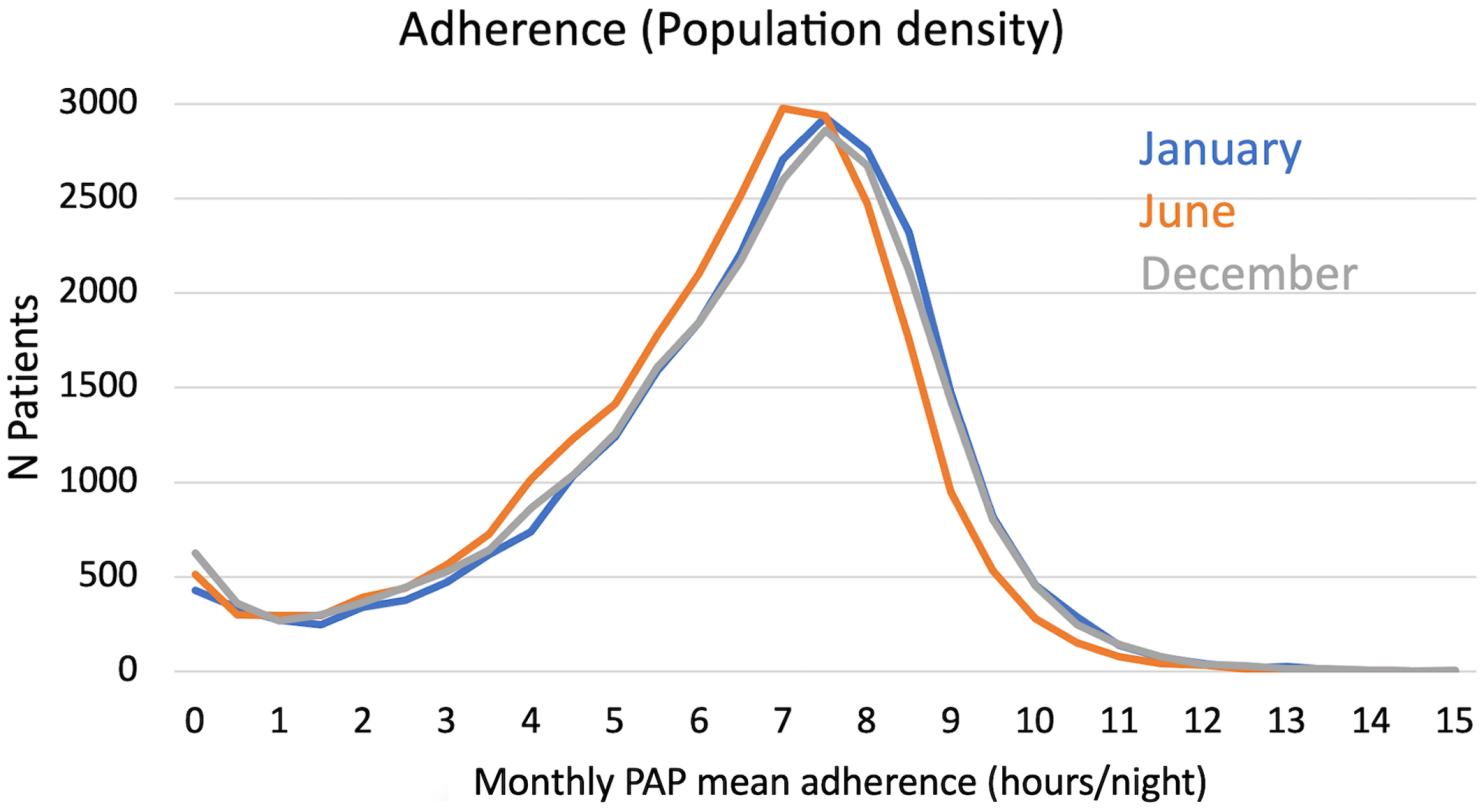
Distribution of monthly positive airway pressure (PAP) mean adherence in January, June, and December.

**Figure 2:**
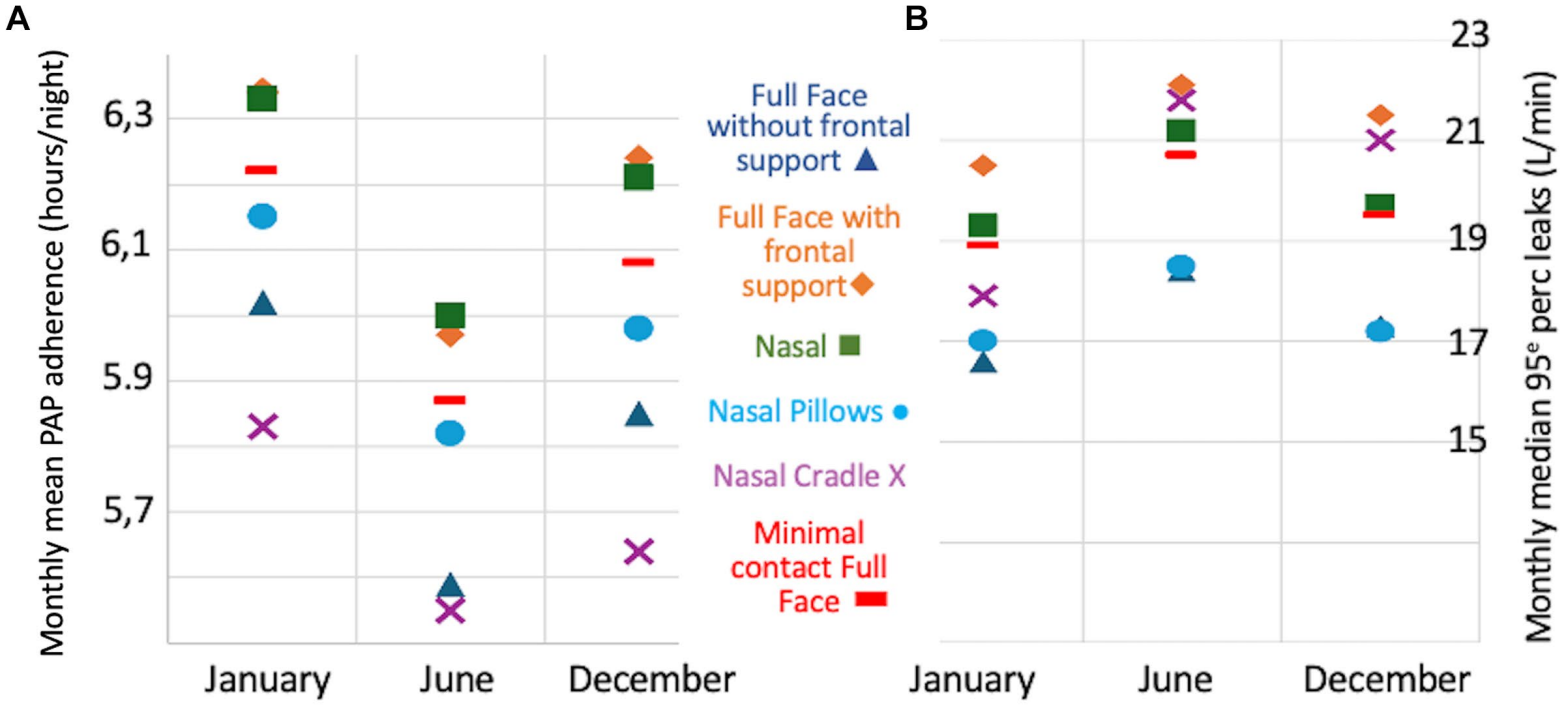
Evolution of positive airway pressure (PAP) adherence [panel **(A)**] and 95th percentile leaks [panel **(B)**] by mask type in January, June, and December.

As illustrated in Figure 1, there was a decrease in PAP adherence in June compared to January and December, with a mean (SD) daily PAP use of 5.87 (2.33) h/night in June compared to 6.22 (2.41) and 6.08 (2.49) h/night in January and December (*p* < 0.001). The mean decrease in daily PAP use between January and June 2021 was 0.37 (1.54) h/night, and the drop in PAP adherence between January and June 2021 achieved the minimal clinically important difference (MCID) of 30 min (1) in 13.9% of adults (*n* = 3,583). As shown in Figure 2, the decrease in daily PAP use in June occurred regardless of the type of mask used and was associated with an increase in non-intentional leaks. The median [IQR] 95th percentile of leaks increased from 15.8 [19.2] and 16.5 [19.4] L/min in January and December to 17.8 [20.3] L/min in June (*p* < 0.001). There was a slight but significant statistical correlation between PAP adherence and 95th percentile non-intentional leaks in June (Spearman’s correlation coefficient: −0.04; *p* < 0.001). Conversely, we found no significant seasonal change in median [IQR] AHI_PAP_ [IQR] (1.38 [2.1], 1.39 [2.1], and 1.32 [2.0] events /h, respectively, in January, June, and December).

**Figure 3:**
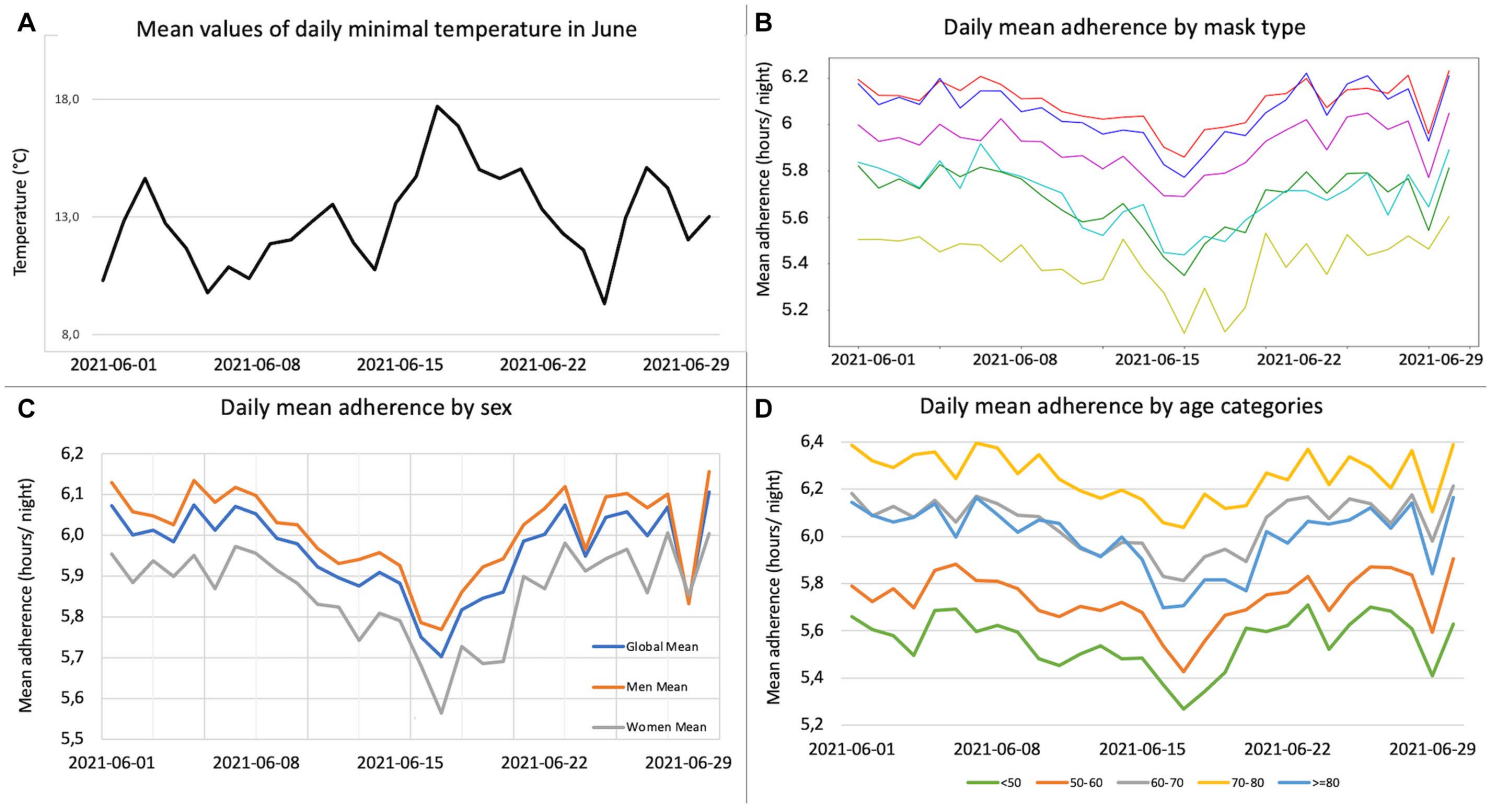
Daily positive airway pressure (PAP) adherence change in June 2021. **(A)** For each day in June, mean of all the minimum daily temperatures for the city (or mean of cities) that best matched the area where each adult lived. **(B)** For each day in June, daily mean adherence by mask type: nasal (red); full face with frontal support (blue); full face without frontal support (green); minimal contact full face (yellow); nasal cradle (cyan); nasal pillows (purple). **(C)** For each day in June, daily mean adherence by sex: general population (blue); men (orange); women (grey). **(D)** For each day in June, daily mean adherence by age categories: under 50 years (green); 50–60 years (orange); 60–70 years (grey); 70–80 years (yellow); 80 years and older (blue).

Further analyses were performed to evaluate the association between daily temperatures and PAP adherence in June 2021. As illustrated in Figure 3, there was an inverse relationship between the evolution of minimal daily temperatures and that of PAP adherence. This trend was observed regardless of mask type, sex, and age category. The association between PAP use and temperature was confirmed by a strong negative Pearson’s correlation coefficient (*r* = −0.57; *p* < 0.001). The lowest values of daily PAP use were observed during the hottest days in June. Mixed linear regression analyses showed a significant association between daily PAP use and temperature (*p* < 0.0001; slope coefficient, −0.07). Conversely, there was no significant association between daily PAP use and AHI and 95th percentile leaks in June (Figure 1).

## Discussion

In a very large sample of 25,846 adults treated with PAP for at least 4 months, the main finding of the present study is a significant decrease in PAP adherence in June compared to January and December. The decrease in PAP use was associated with rising temperatures and achieved the 30-min MCID for 13.9% of adults.

It can be hypothesized that changes in sleep duration might have contributed to the decline in PAP adherence in June compared to January. Several studies have demonstrated that sleep duration is longer in winter and shorter in summer, likely due to increased day length and/or increased temperature, with the effects being particularly pronounced in children or the elderly (3). The production of melatonin, a sleep-promoting hormone, is linked to light exposure; more light inhibits melatonin production, while less light increases melatonin production (4). A telephone survey of Maryland residents reported that the average sleep duration during the winter was 7.41 h, compared with 7.05 h during the summer (5). Using questionnaires including items on sleep duration and sleep problems, Suzuki et al. demonstrated seasonal changes in sleep duration in 1,388 Japanese community residents aged 15–89 years, with the longest in winter and the shortest in summer (winter–summer difference: 11.4 ± 1.8 min) (6). Using wearable devices in 216 individuals across the U.S., Mattingly et al. demonstrated a 25-min decrease in sleep duration in spring compared to winter (3). The seasonality of human sleep has also been demonstrated using polysomnographic data, with a maximum difference in monthly mean TST of 62 min between January and June (4). Our finding that PAP use decreases in June compared to January is consistent with a previous report from Fujino and al. (7) showing a decrease in nightly PAP use from 356 ± 86 min in winter to 329 ± 81 min in summer in 141 patients treated with PAP for >12 months in Japan (difference of day length between January and June in Japan (Tokyo): 4 h34; in France (Paris): 7 h27). The authors also reported modest but statistically significant changes in AHI_PAP_, which were not confirmed by our study. On the other hand, our study shows a significant increase in mask leaks in June. The fact that changes in leaks were uniform regardless of mask type suggests a common factor across all masks rather than one related to the mask type. We hypothesize that the observed leak increase could be the consequence of poorer sleep quality in June, with mask displacement and/or perspiration inducing a loss of the seal of the mask cushion.

There is increasing evidence that rising temperatures erode human sleep globally (8), especially among the most vulnerable populations, including the elderly and those with low income. Using billions of repeated sleep measurements from sleep-tracking wristbands across 68 countries linked to local daily meteorological data, Minor et al. (9) demonstrated that increased temperature shortens sleep primarily through delayed onset, increasing the probability of insufficient sleep. Our study provides novel data supporting the contribution of rising temperatures to seasonal changes in PAP use. As observed before (10), the most pronounced decline in PAP adherence was observed during the hottest days, while PAP adherence was only slightly reduced during the coolest days of June. These variations have been observed while day length is stable during June, thus supporting the fact that temperatures might be associated with the decrease in PAP use.

Some limitations should be taken into account when interpreting our study. Being fully based on telemonitoring data, several factors that may influence PAP adherence, such as socioeconomic status, OSA severity, and comorbidities, were not taken into account in the analysis. We also acknowledge that sleep duration was not measured, thus its influence on PAP duration was not measured in the present study.

Clinicians should be aware of seasonal changes in PAP adherence in adult patients with OSA. Rising temperatures were associated with a decrease in PAP adherence in June. Patients should be advised to keep their bedroom as dark and cool as possible. Moreover, PAP telemonitoring must take these data into account to adapt the adherence alert threshold during the spring and summer. Seasonal changes in PAP use are likely to be exacerbated by climate change.

## Data availability statement

The raw data supporting the conclusions of this article will be made available by the authors, without undue reservation.

## Ethics statement

Ethical approval was not required for the study involving humans in accordance with the local legislation and institutional requirements. Written informed consent to participate in this study was not required from the participants or the participants’ legal guardians/next of kin in accordance with the national legislation and the institutional requirements.

## Author contributions

AP: Conceptualization, Formal analysis, Writing – original draft, Writing – review & editing. CB: Conceptualization, Formal analysis, Investigation, Writing – original draft, Writing – review & editing. DJ: Supervision, Writing – review & editing. AS: Data curation, Methodology, Writing – review & editing. FB: Data curation, Investigation, Writing – review & editing. FG: Supervision, Writing – review & editing.
